# Sustained fluvial deposition recorded in Mars’ Noachian stratigraphic record

**DOI:** 10.1038/s41467-020-15622-0

**Published:** 2020-05-05

**Authors:** Francesco Salese, William J. McMahon, Matthew R. Balme, Veronique Ansan, Joel M. Davis, Maarten G. Kleinhans

**Affiliations:** 1 Faculty of Geosciences, Utrecht University, Princetonlaan 8a, Utrecht, 3584 CB The Netherlands; 2 International Research School of Planetary Sciences, Università Gabriele D’Annunzio, Viale Pindaro 42, Pescara, 65127 Italy; 3 Planetary Environments Group, Open University, Walton Hall, Milton Keynes, UK; 4 LPG Nantes, UMR6112, CNRS-Université de Nantes, 2 rue de la Houssinère, BP 92208, 44322 Nantes Cedex 3, France; 5 Department of Earth Sciences, Natural History Museum, Cromwell Road, Kensington, London, SW7 5BD UK

**Keywords:** Inner planets, Geomorphology

## Abstract

Orbital observation has revealed a rich record of fluvial landforms on Mars, with much of this record dating 3.6–3.0 Ga. Despite widespread geomorphic evidence, few analyses of Mars’ alluvial sedimentary-stratigraphic record exist, with detailed studies of alluvium largely limited to smaller sand-bodies amenable to study in-situ by rovers. These typically metre-scale outcrop dimensions have prevented interpretation of larger scale channel-morphology and long-term basin evolution, vital for understanding the past Martian climate. Here we give an interpretation of a large sedimentary succession at Izola mensa within the NW Hellas Basin rim. The succession comprises channel and barform packages which together demonstrate that river deposition was already well established >3.7 Ga. The deposits mirror terrestrial analogues subject to low-peak discharge variation, implying that river deposition at Izola was subject to sustained, potentially perennial, fluvial flow. Such conditions would require an environment capable of maintaining large volumes of water for extensive time-periods, necessitating a precipitation-driven hydrological cycle.

## Abstract (web summary)

Using high-resolution orbital imagery of the Martian surface, the authors Salese et al. here describe the first discovered stratigraphic product of multiple extensive fluvial-channel belts in an exposed vertical section at Izola Mensa in the northwestern rim of the Hellas Basin.

## Introduction

While the present-day Martian surface is generally dry and cold, its geomorphic record contains compelling evidence for the former presence of liquid water^1–7^. In addition to this rich geomorphic archive, Mars’ increasingly accessible sedimentary rock record provides a repository of information from which to study how planet-wide patterns in deposition have changed over time. From orbit, Martian sedimentary rocks have been observed for more than 20 years^8–13^, but detailed descriptions of large Noachian alluvial successions have so far been lacking. In fact, unequivocal sedimentary-stratigraphic evidence of alluvium has only been identified in-situ by rover-led studies^14^, and whilst rovers provide unprecedented direct access to extraterrestrial strata^15^, methodologies are typically limited by accessible outcrop dimensions. For example, the Shaler outcrop, an interpreted Hesperian fluvial deposit identified by Curiosity^16^, comprises a single 70-cm-thick, 20-m-wide sandstone body. This is a crucial scale difference when compared with orbital investigation: the physical dimensions of most rock outcrops studied on the ground to date are smaller than the dimensions of many geomorphic components of even moderate-sized extant river systems^17,18^.

**Fig. 1:**
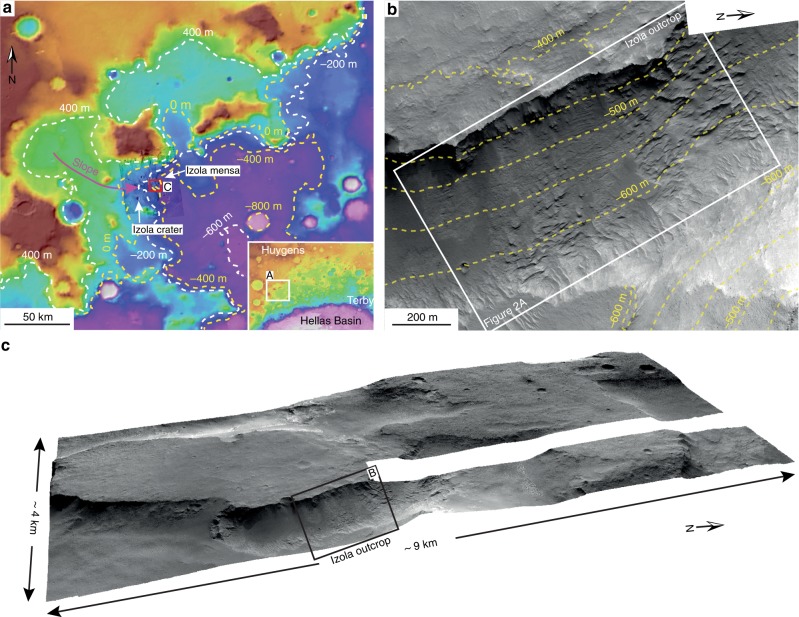
General context of study location at Izola mensa. **a** Mars Orbiter Laser Altimeter (MOLA) topographic map centred on the NW rim of the Hellas basin, showing a depression (blue/violet) in the centre-lower right corner, the cratered highlands (brown-orange) in the upper left corner and the location of the studied outcrop within the red box. The magenta arrow indicates the slope direction of the modern surface. The identified macroforms suggest that the outcrop is broadly cut in the depositional-strike direction, in alignment with regional slope (**a**). The topographic elevation spans from 1500 m (brown) to −1350 m (white). Bottom right inset shows the regional context. Mars Orbiter Laser Altimeter (MOLA) topographic map centred on the northwestern shoulder of the Hellas basin. Study area indicated by white box. The topographic elevation spans from 4319 m (brown) to −8194 m (white). **b** High-resolution imaging science experiment (HiRISE) contour lines from HiRISE digital terrain model (1 m/pixel) (HiRISE stereo pairs ESP_055357_1540; PSP_003799_1540) over HiRISE visible image (25 cm/pixel). The Izola outcrop is located at 25.88°S and 54.29°E and faces almost N-S. **c** 3D view without vertical exaggeration of the Izola outcrop shown in **b**. HiRISE ESP_055357_1540 (25 cm/pixel) draped on HiRISE digital terrain model (1 m/pixel).

In this study, we use high-resolution imaging science experiment (HiRISE) image (25 cm/pixel) and topographic (1 m/pixel) data to describe the sedimentary-stratigraphic architecture of a far-larger, 1500-m-wide, 190-m-thick sedimentary succession. The recently identified Izola outcrop is located in the northwestern rim of the Hellas basin (Fig. 1a), a ~2000 km diameter impact structure containing a variety of 3.7 Ga Noachian Fe/Mg phyllosilicate-rich sedimentary intercrater plains, overlain by Hesperian-aged (~3.3 Ga) lava flows^19^.

These intercrater plains offer erosional windows which expose stratigraphic sections with well-preserved channel forms, and which must be older than the ~3.7 Ga overlying plains^19^. The channel forms and associated sedimentary packages are interpreted as the product of an actively depositing fluvial system, with the final sedimentary architecture suggesting that these Noachian-aged rivers were not typified by high-energy episodic floods, but rather perennial or semi-perennial fluvial flow. The scale and completeness of the sedimentary succession offers a so far unique opportunity to assess the larger scale morphology of an evolving Noachian-aged fluvial system.

## Results and discussion

### The sedimentary succession

The outcrop exposes layered sedimentary strata, which display a variety of large-scale stratal architectures consistent with an alluvial interpretation. Alluvial sedimentary strata can be subdivided into genetically related three-dimensional packages^20–22^. The aggregate of these packages is referred to as the succession’s sedimentary architecture, and is the product of the scale and behaviour of the fluvial system over time. The outcrop appears to have undergone little post-depositional deformation, has a gentle dip and large-dimensions, so is suitable for the analysis of sedimentary architecture (Supplementary Fig. 5). A hierarchy of bounding surfaces is applied to divide stratigraphy, hierarchically ordered to reflect river processes at varying scales (Table 1, Supplementary Fig. 1). Two distinct sedimentary packages were recognised in the studied outcrop: (1) channelised packages; and (2) inclined accretionary surfaces. As details of sedimentary facies (usually discriminated by grain size and centimetre to decimetre-scale bedding and sedimentary structure) are unattainable without in-situ investigation on the ground, only the three-dimensional geometry of the sedimentary packages are described, with no details of internal structure attempted.

**Table 1:** Hierarchical division of bounding surfaces applied in this study.

Bounding surface order	Maximum lateral extent	Bounded units	Sedimentary process
Fifth	640 m	Channel belts	Switch between dominant depositional process (e.g., channel-belt avulsion)
Fourth	210 m	Channel fills, fluvial barforms	Termination of accretion or reworking of a discrete macroform
Third	180 m	Macroforms	Accretion of a discrete macroform
Second	Not observable (rover only)	Cosets	Accretion and reworking of mesoforms
First	Not observable (rover only)	Sets	Ripple or dune migration
Zeroth	Not observable (rover only)	Laminae	Burst-sweep cycle

Bounding surface ranking and process interpretations adopted from previous studies^22,62^.

### Channelised packages

**Fig. 2:**
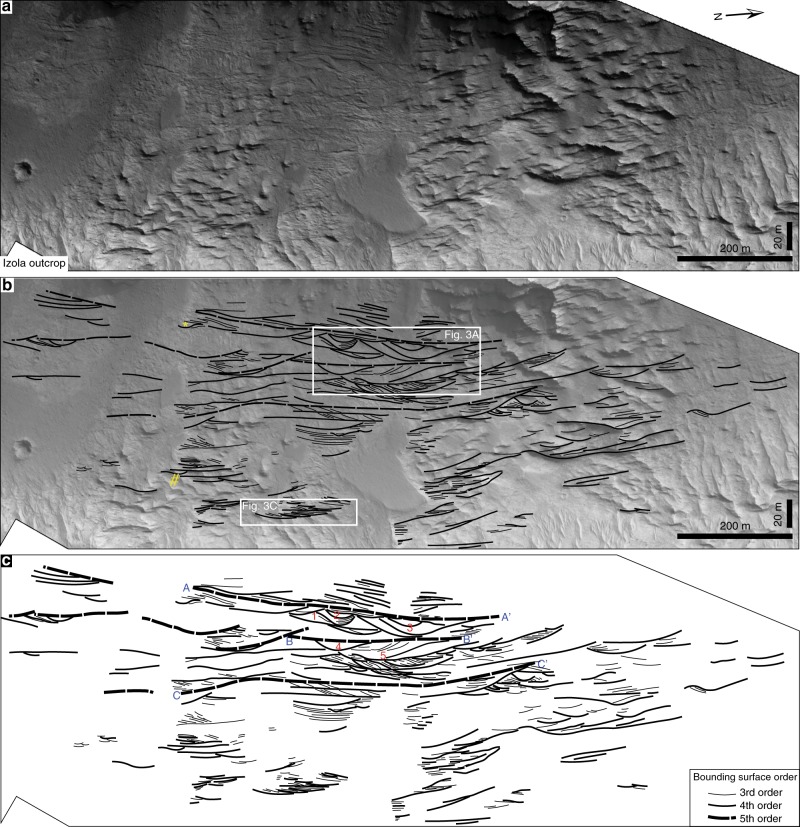
Architectural analysis of Martian channel forms and inclined accretionary surfaces at the Izola outcrop. **a** High-resolution imaging science experiment (HiRISE) image of the studied outcrop (ESP_055357_1540; 25 cm/pixel resolution). Objects down to 77 cm can be resolved. The outcrop is 1500-m-wide and 190-m-thick. Note the different scale between the vertical and horizontal axis (vertically exaggerated). **b** Line drawing of **a** to illustrate an architectural interpretation, displayed in **c**. Line drawings were only attempted in areas where stratigraphy was clearly visible (no lines were joined across areas on non-exposure). The yellow asterisk and hashtag link to Supplementary Figs. S3 and S4, both possible examples of channel wings. Identified fifth-order surfaces are labelled with letters and surface dimensions listed in Table 2. The well-exposed channels are labelled with numbers and their parameters reported in Table 2.

**Fig. 3:**
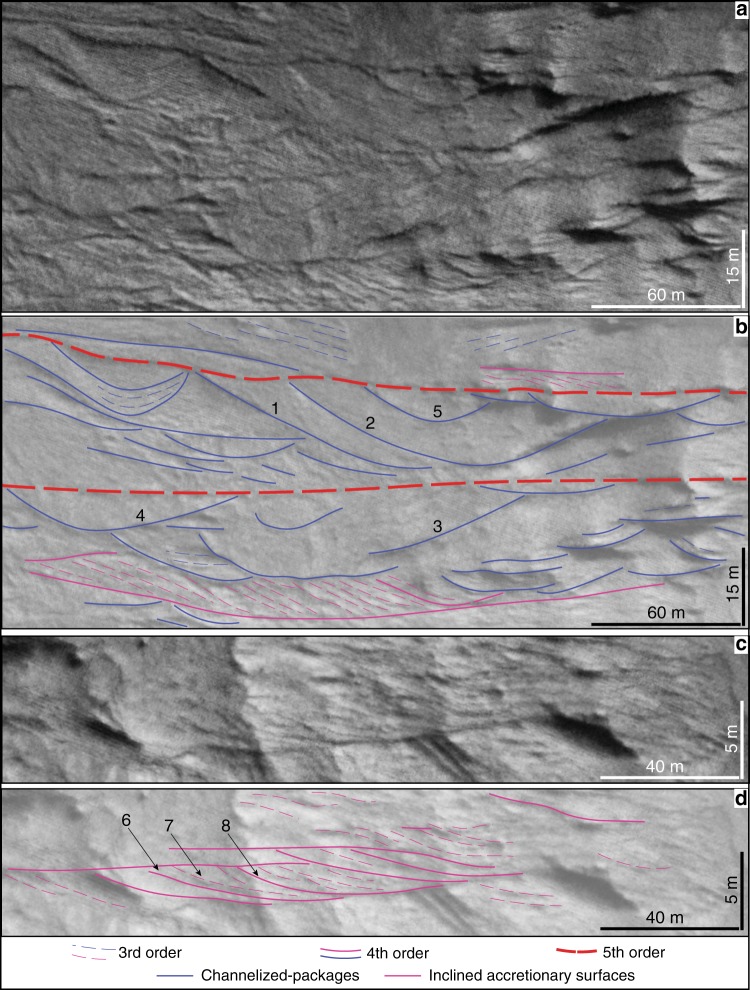
Example channelised packages and inclined accretionary surfaces. **a** Close up of white box indicated in Fig. 2b. **b** Architectural interpretation of **a** displaying a number of channels (blue lines). Some examples preserve former channel margins, strong evidence for original channel lateral migration. Channelised packages have associated inclined accretion surfaces (magenta lines), with all packages topped and floored by more extensive fifth-order surfaces (red lines). Fifth-order surfaces possibly archive avulsion events. A previous channel margin (1 + 3) laterally coalesces with the final channel margin (2 + 4) indicating that the original active channel migrated laterally. A nested channel-cut (5) is present within a larger channelised package. **c** Close up of white box indicated in Fig. 2b. **d** Examples of inclined accretion surfaces with distinct foreset and bottomset elements (topsets truncated by overlying strata). Downlapping of internal, third-order surfaces suggest multiple accretion phases. Note that line drawings are not attempted in areas where exposure is poor. A third-order surface (7) downlaps the bottomset of an underlying fourth-order surface (6) indicating bar migration. A subsequent fourth-order surface (8) truncates the previous fourth-order surface (6) indicating multiple phases of bar-building activity.

In the observed stratigraphy, packages bound by lower erosional, channel-shaped (fourth order) surfaces and truncated by flat, erosional (fourth and fifth order) surfaces are attributed to channel-fill deposition (Figs. 2, 3b, Supplementary Fig. 2A). They are 5–15 m thick, with observable lateral extents of up to 210 m (although outcrop limitations restrict observation of the full lateral extent of many channel forms: Table 2, Supplementary Fig. 2). Internally, packages appear succession-dominated^23^, comprising multiple aggrading (third order) surfaces (Fig. 3b). Final channel banks and former channel margins coalesce, indicating that the original channels laterally migrated (Fig. 3b, d). Some channelised packages have a distinct channel wing (yellow asterisks in Fig. 2b; Supplementary Figs. 3, 4), which may archive a genetically associated levee or crevasse and thus strengthen the alluvial interpretation. Particular areas of the outcrop show high concentrations of discrete, but partly amalgamated, channelised packages (Fig. 3b). In these areas, smaller channelised packages may be nested inside larger examples (Fig. 3b), implying that periods of net erosion locally occurred within a dominantly depositional regime. Areas containing clusters of channels are bottomed and topped by laterally extensive (up to 640 m), low relief, fifth-order surfaces, which suggest a change in the type or location of the dominant depositional process. These surfaces probably reflect channel avulsion, in which the location of the active channel changes abruptly. The sediments enclosed between these fifth-order surfaces were therefore laid down between avulsion events and are thus defined as channel belts^24^.

**Table 2:** Fourth and fifth order observed bounding surface dimensions.

	Depth (m)	Width (m)	Width/depth ratio
Fourth-order ID			
1	8	150	18
2	11	110	10
3	15	210	14
4	7	110	15
5	15	170	11
Fifth-order ID			
A–A′	Undefined	570	N/A
B–B′	21	345	N/A
C–C′	30	640	N/A

The ID locations of these bounding surfaces are reported in Fig. 2c.

### Inclined accretionary surfaces

In the observed stratigraphy, wedge-shaped packages comprise gently inclined depositional surfaces (third and fourth order; Fig. 3d). They are topped and bottomed by flat, erosional surfaces (both fourth order), except on occasions where they can be traced laterally into an associated channelised package (Fig. 3b). Most frequently only erosional remnants are preserved, with deposits often passing laterally into areas of non-exposure (Fig. 3d). Thicknesses range from 1.5 to 14 m, similar to the associated channels. Geometry and internal stacking patterns are suggestive of shifting fluvial barforms. For example, in some instances, bottomsets of fourth-order surfaces are downlapped by third-order foresets (Fig. 3d), demonstrating discrete intervals of bar-building. The outcrop orientation with respect to paleoflow is not well known, meaning it is not possible to build any unequivocal consensus on the direction of barform accretion with respect to the flow of the original channels (e.g., downstream accretion, lateral accretion). However, the identification of channel forms in conjunction with the outcrops alignment with the regional slope direction suggests a broadly depositional-strike succession.

### Alternative explanations

Without in-situ validation of fluvial deposition other alternative possibilities must additionally be considered. In this section, we examine two alternative origins for the studied sedimentary succession: aeolian deposition, and deposition within submarine channels.

Aeolian: if these deposits have an aeolian origin, then the working hypothesis is that the channel forms are in fact localised scour fills formed by migrating aeolian strata, and that this succession records periods of aeolian accumulation punctuated by erosion or stagnation. However, there are disconnects between the stratigraphic architecture described here, and that of large-scale cross-bedding typical of aeolian bedforms described on both Earth^25,26^ and Mars^27^. No aeolian interpretation can explain the observable lateral migration surfaces associated with the discrete channelised packages (Fig. 3b). Multiple generations of aeolian dunes would partially erode preceding examples, perceived at outcrop by marked downlapping surfaces not apparent anywhere across the succession. Also, the fill geometry of aeolian dune deposits is often symmetrical and concordant with the trough base, unless the final outcrop is cut obliquely to the original flow direction, in which case planes are perceived to fill asymmetrically and downlap onto the trough base^28^ (again, an architectural style not recognised here). Finally, multiple possible examples of channel wings, evident from a distinct inflexion point, are found across the outcrop (Fig. 2b, Supplementary Figs. 3, 4). Such elements can be variably interpreted as the topmost story of an individual channel or a genetically associated levee or crevasse^23^, but do not concur with any aeolian model.

Submarine channels: certain depositional structures are found in both submarine and alluvial environments (e.g., channel wings), but several architectural characteristics in these deposits favour alluvial interpretation and suggest against a submarine origin. Evidence of lateral migration is widespread across the outcrop (i.e., inclined accretionary surfaces). Such an architectural style is more typical of alluvial settings, with submarine slope architectures more regularly dominated by vertical accretion^29^. Some inclined accretion surfaces additionally have distinct foreset and bottomset elements (Fig. 3d), consistent with those of fluvial barforms which scale to bankfull water-depth. Furthermore, most described submarine slope channels on Earth are at least an order of magnitude greater in size than the channels identified in this study^30^, which have dimensions consistent with many other geomorphic fluvial channels identified across the Martian surface^31^. Finally, regional observations of paleolakes occupying the Hellas basin at various stages in its evolution^32^ are compatible with an alluvial interpretation for these deposits.

Although a degree of ambiguity exists in any interpretation of ancient sedimentary strata, whether on Earth or Mars, and disputes between subaqueous and subaerial interpretations are commonplace on both planets^10,33,34^, considering the discussion herein, we argue that outcrop evidence strongly favours a fluvial origin for the described Izola architecture.

### Depositional environment

From outcrop measurements, the channel and barform thicknesses are up to 13 and 14 m, respectively. This provides minimum constraints for bankfull water-depths at the time of deposition. Numerical models demonstrate that preserved sand-body thickness is on average 30% of the original channel depth^35^, so this approximation is almost certainly an underestimate. These hypothesised water-depths would be comparable with those of modern, mature fluvial systems on Earth draining extensive basins^36^, suggesting these Noachian-aged rivers had similar drainage capacities.

Channels and barforms account for the entire observable stratigraphy. The evidence for channels being succession-dominated (Fig. 3b) implies that entrenchment was quickly followed by bed aggradation^23^. The occurrence of distinct channel clusters is consistent with channel belts with relatively stable banks and limited lateral mobility^37^. The internal structure of the identified barforms comprises remnants of numerous phases of bar-building activity (Fig. 3d). On Earth, deposits of rivers with low-peak discharge variability preserve macroform structure, enabling the reconstruction of barform morphology^35,38^. The similar preserved architectural style seen here, of relatively intact barforms and channels, implies that the depositing Izola rivers were also characterised by low-peak discharge variability. Episodic flooding events may not have been responsible for deposition, but rather long-term, potentially perennial fluvial flow. This conclusion also indicates that the Izola rivers were not the product of meltwater flowing from a glacial front, as these environments are more widely associated with fluctuating, high discharges, and so would be unlikely to leave a stratigraphic record comprising relatively intact channels and bars.

### Siliciclastic deposition on an unvegetated planet

Depositional models are largely based on sedimentary environments on Earth, where physical form and process is near ubiquitously influenced by biology^39^. Comparisons between the terrestrial and Martian sedimentary record therefore require careful consideration before application. For instance, copious observations demonstrate the various ways extant vegetation modifies fluvial processes and landforms^40,41^. Most important from an astrobiological standpoint is the likelihood of alluvial mud becoming preserved on an unvegetated planet. In addition to increasing mud production through chemical weathering^42^, terrestrial vegetation promotes mud retention on the continents through above-ground baffling and below-ground stabilisation^41,43,44^. Through these combined processes, an upsurge in mudstone abundance within alluvial stratigraphy is observed in stratigraphic alignment with evolving land plants^45^. Before this time, terrestrial alluvium is predominantly sand-grade or coarser with few preserved muddy floodplain facies^45,46^. Direct comparison between the pre-vegetation Earth and unvegetated Martian record might therefore imply that the studied Izola alluvium might also be lacking in preserved mudstone. Such tangible characteristics of the terrestrial pre-vegetation alluvial record have been used previously as supportive evidence for environmental interpretations elsewhere on Mars: for example, the scarcity of pre-vegetation terrestrial floodplain mudstones was used to recommend a lacustrine, not floodplain, origin of the Sheepbed mudstone at Gale crater^16^. The abundance of mudstone in the studied outcrop here is not known or speculated upon, as their intrinsic fine-grained components require rover-based observations. However, the preserved channel-belt architecture, comprising relatively intact channels and barforms, suggests some degree of original channel-belt stability, with naturally shear-resistant sediment such as mud a possible candidate^47,48^. With regard to channel and barform facies, evidence of relatively stable, deep-channelled drainage on pre-vegetation Earth is being increasingly reported^37^. Such findings are helping to dispel notions that pre-vegetation rivers were ubiquitously wide and shallow^49^, an observation that can now be extended to Mars (Fig. 2).

### Preservation of time and implications for early Mars climate

Disentangling the total duration recorded in any sedimentary outcrop is difficult as stratigraphic records are highly fragmentary, incomplete chronicles of time^50,51^. Despite uncertainty, broad approximations from the studied outcrop can be made which can inform ongoing debates about the early Martian climate^52^. The outcrop comprises at least four possible channel belts (Fig. 2), discrete packages of strata bound by laterally extensive (fifth order) surfaces and, on Earth, channel belts of this scale can require up to ~10^4^–10^5^ terrestrial years to deposit^52^ (though we recognise that this approximation may differ under Mars’ distinct boundary conditions). However, the amount of additional time hidden within the bounding fifth-order surfaces between individual channel belts is unknown (and unknowable)^53^. If a channel-belt interpretation is correct, the laterally extensive surfaces developed after the active channel-belt was transposed through avulsion. During this time, deposition was likely occurring elsewhere in the basin, and the region covered by our sedimentary outcrop was undergoing intervals of net erosion or stasis^54^. In other words, hiatuses in deposition in the studied outcrop were accompanied by deposition elsewhere in the basin. This implies that the time recorded in the 190-m-thick succession represents only a fraction of the total time fluvial deposition was ongoing in this region. The majority of strata and time will have either been lost to erosion, or preserved in outcrops as of yet undiscovered, or currently buried and not amenable to study. While we are only beginning to understand the chronostratigraphic exactness of sedimentary rock outcrops on Earth^53,55,56^, let alone Mars, it appears likely that the period of deposition in the northwestern rim of the Hellas basin exceeded 10^5^ terrestrial years. Furthermore, the preservation of relatively intact channel margins and barforms advocates that throughout this protracted period fluvial deposition was a relatively constant phenomena^38^.

The architectural interpretation of this so far unique sedimentary succession feeds into ongoing debates about the early Martian climate. Our interpretation of long-lived, deep, perennial or semi-perennial rivers necessitates a climate in which active water-conduits were maintained for 10^5^ years or longer. For the first time, orbital data has allowed us to examine, through detailed high-resolution architectural analysis, a large (1500 m by 190 m) pre-late Noachian outcrop, and draw reliable paleoenvironmental interpretations based on sedimentary-stratigraphic evidence. Our observations and analysis favour steady water discharges that are most consistent with a precipitation-driven hydrological cycle. This conclusion aligns with previous arguments for the prolonged presence of water on the early Martian surface drawn from alternative geomorphological^2,6,14,19^ and mineralogical^57,58^ observations.

## Methods

### DTM construction

High-resolution imaging science experiment^59^ image ESP_055357_1540 acquired in May 2018 along with HiRISE image PSP_003799_1540 (acquired in 2007) enabled the construction of a centimetre scale DTM. Both images have 25.6 cm/pixel (with 1 × 1 binning) resolution so objects down to 77 cm can be resolved. A digital terrain model (DTM) was produced from the HiRISE images ESP_055357_1540 and PSP_003799_1540 using the USGS Integrated Software for Imagers and Spectrometers (ISIS) software and the BAE photogrammetric package SOCET SET according to a previously used methodology^60^. Tie points were automatically populated in SOCET SET between the two images. We ran a series of bundle adjustments, removing erroneous tie points until the remaining points had an RMS pixel matching error of ≤0.6 pixels. The resultant DTM was then tied to Mars Orbital Laser Altimeter^61^ topography and exported with a horizontal post spacing of 1 m/pixel and a vertical precision of ~1 m.

### Calculation of sedimentary package dimensions

The acquired HiRISE DTM of the Hellas outcrop was of sufficient resolution to enable accurate tracing of beds and plotting of architectural elements (Fig. 2). Channels thickness were obtained measuring the exact elevation of the channel top and the exact elevation of the channel base using ArcMap 10.6 elevation tools. Channel widths were measured by tracing an edge-to-edge topographic channel profile using the HiRISE DTM. This allowed calculation of true thickness, given that they are bound by laterally extensive fifth-order surfaces, which are almost flat lying (Supplementary Fig. 5).

### Architectural analysis

Line drawings were only attempted at areas where stratigraphy is clearly visible. Outcrop orientation with respect to paleoflow is not known, so no architectural elements with distinct directional components (e.g., downstream accretion, lateral accretion) were assigned. Sediment grain size is also unknown, thereby prohibiting the distinction between active and abandoned channel-fill deposits. The completed architectural panel enabled the various sediment stacking patterns and lateral relationships assessed.

### Bounding surface hierarchy

A hierarchy of bounding surfaces was used to describe partitions of fluvial strata at outcrop, with different order surfaces reflecting river processes at varying scales^62^ (Table 1, Supplementary Fig. 1). The principles of the hierarchical division applied here follows that detailed in previous papers^22,62^. Succinctly, zeroth, first and second order surfaces relate to foreset, set and coset boundaries, respectively, and are not observable from HiRISE imagery. Third- and fourth-order surfaces indicate the presence of macroforms (e.g., a barform deposit) or a channel. Fourth-order surfaces represent the upper and lower boundaries of the macroform or channel, whereas third-order surfaces relate to internal growth increments (indicating flow fluctuation, but no significant changes in predominant fluvial style). Fifth-order surfaces are the highest order observed at the studied outcrop and bind major depositional packages (e.g., channel belts). More fifth-order surfaces are likely present in the studied outcrop than highlighted on Fig. 2. This is simply because their confident recognition depends on an understanding of their relationship with lower order surfaces, and in some instances the vagaries of outcrop exposure (particularly towards the bottom of the section) prevent this.

### Estimation of outcrop age

The age of the intercrater plains, which form the planform cover of the studied sedimentary-stratigraphic succession, has been estimated in a previous work^19^ using various crater count techniques. They date 3.70 + 0.03/−0.04 Ga (Noachian). The latter represents the age of the surface at the top of the outcrop studied in this work, which lies in the S1 unit (Fig. 18^19^). Crater counts were performed on Context Camera (CTX; 5–6 m/pixel) data using Crater Tools. Crater statistics and crater model ages were analysed with Craterstats2 software. For more details and references see a previous study^19^.

## Supplementary information

Supplementary Information

## Data Availability

The HiRISE data that support the findings of this study were obtained freely from the Planetary Data System (PDS) and are publicly available online at https://pds.nasa.gov/index.shtml. Satellite imagery and the Extended Data were generated with ISIS 3 (Integrated Software for Imagers and Spectrometers) available online at https://isis.astrogeology.usgs.gov. All these data were integrated into ArcMap 10.6 project. The DTM was produced using the USGS Integrated Software for Imagers and Spectrometers (ISIS 3) software and the BAE photogrammetric package SOCET SET with a post spacing of 1 m/pixel.
