# Supplementary material for: Sustained fluvial deposition recorded in Mars’ Noachian stratigraphic record
Source: Nat Commun. 2020 May 5;11:2067. doi: 10.1038/s41467-020-15622-0 (PMC7200759; doi:10.1038/s41467-020-15622-0)
Supplement: Supplementary file 1 — Supplementary Information [file 41467_2020_15622_MOESM1_ESM.pdf]

Supplementary Information

SUSTAINED FLUVIAL DEPOSITION RECORDED IN MARS' NOACHIAN STRATIGRAPHIC RECORD

Salese et al.

___

Supplementary Figures

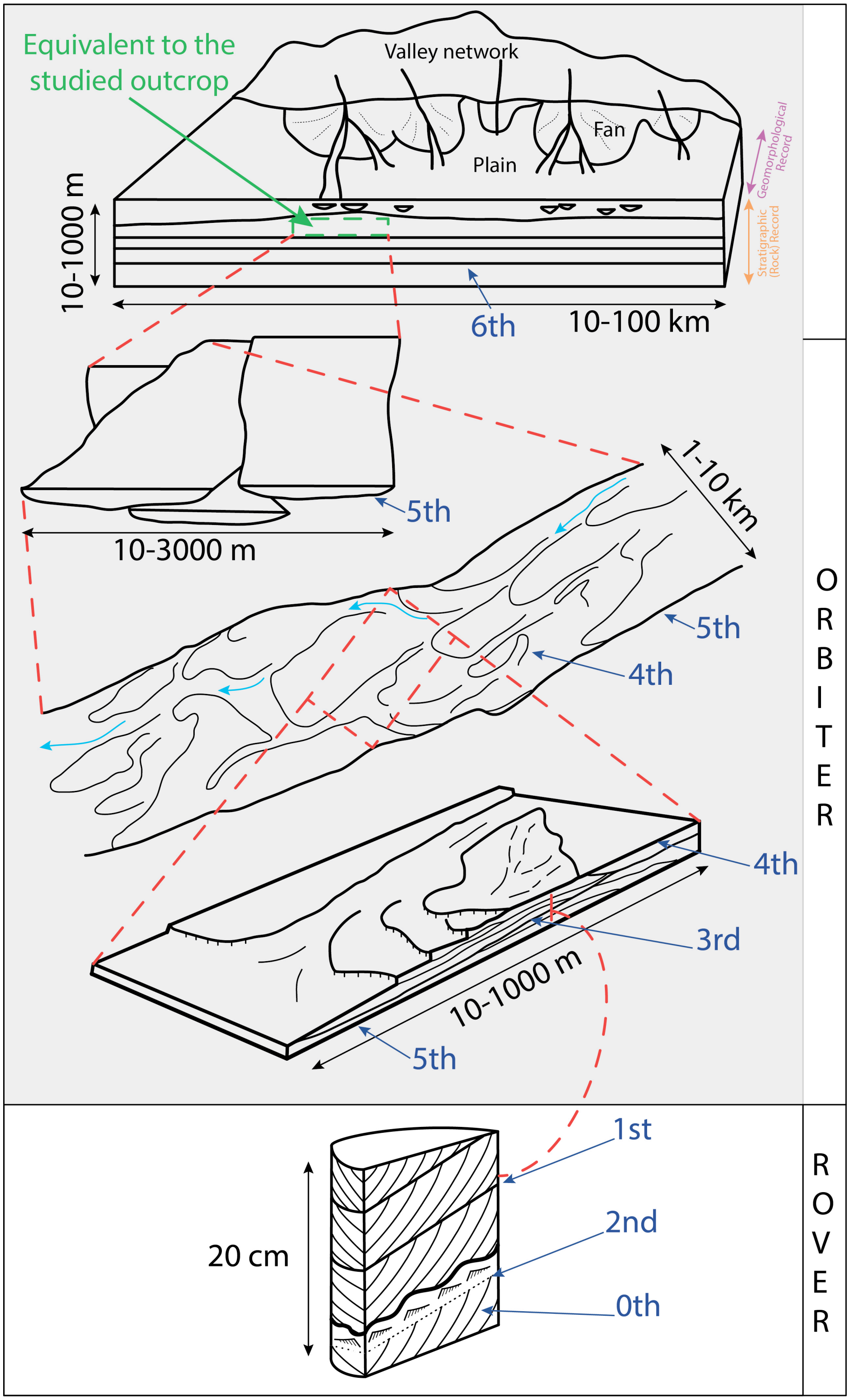

Supplementary Figure 1. Hierarchy of fluvial depositional units amenable for study on Mars by rover and orbiter technology. Modified from Miall 1985, see main text for reference. Bounding surfaces identifiable through rover

technology (0th to 3rd order) and from orbit (3rd to 5th order). In this paper bounding surfaces ranked from 3rd to 5th order have been identified. The lateral dimensions of the outcrop do not enable identification of extensive 6th order-surfaces. In the sketch at the top of this image the location of an equivalent outcrop to that studied is shown (see green text and box). Most previous martian fluvial analyses have been made using the planet's geomorphic record (represented in the sketch at the top of this image and indicated by the violet arrow and text). This geomorphic record contains compelling evidence for the former presence of water (e.g., valley network). Nevertheless, a complementary repository of information comes from the planets sedimentary-stratigraphic record (green box in top sketch). Although these two datasets, the geomorphic in planform and the sedimentary-stratigraphic in section, lie in the same geographic context (e.g., the valley network in the northern rim of Hellas and the studied outcrop), they are not direct equivalents because the geomorphic record (e.g., valley network) represent only the final snapshots of deposition, whereas the sedimentary-stratigraphic record, and hence the outcrop studied in this work, represent a sequence of events that occurred over a more prolonged period.

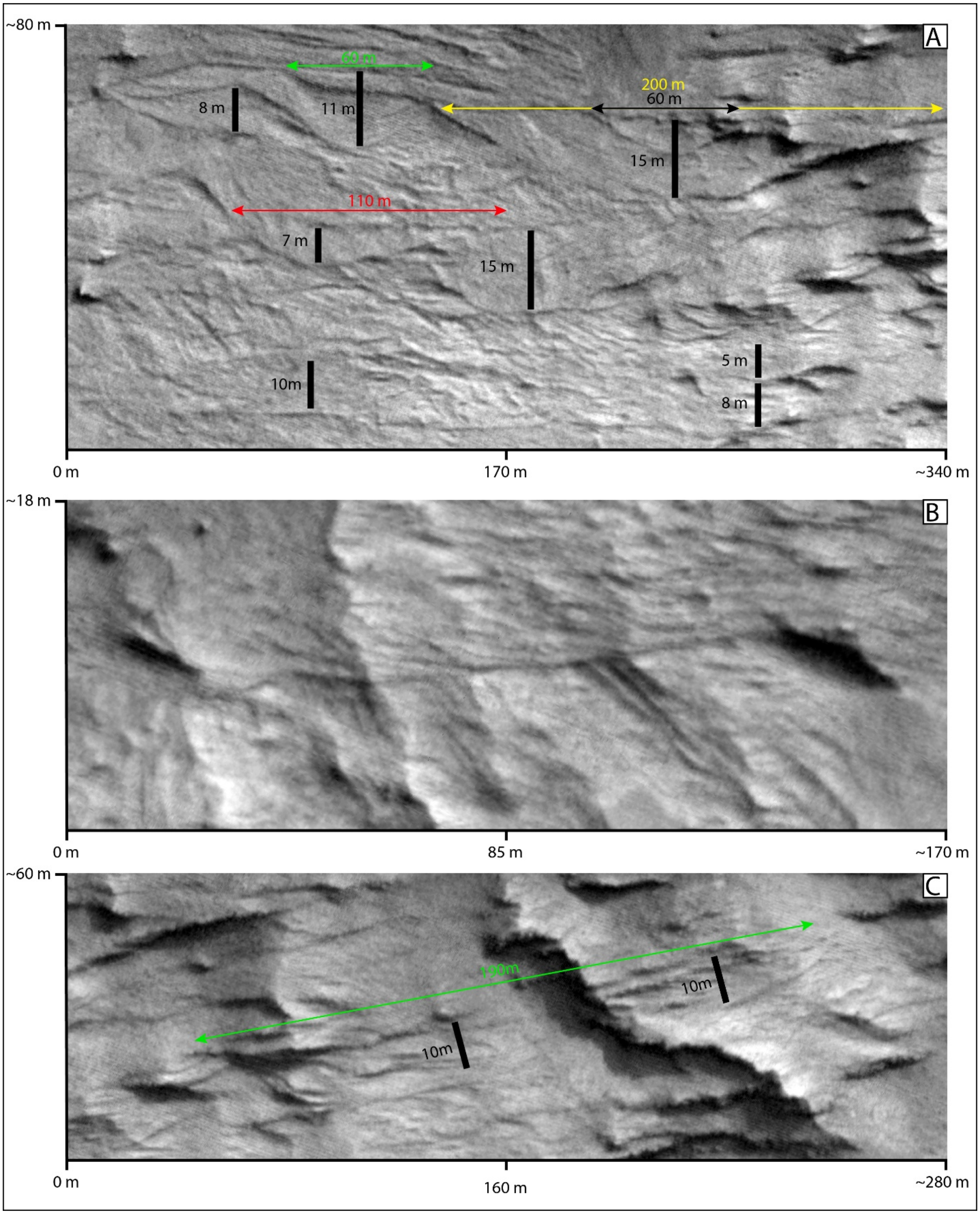

Supplementary Figure 2 The dimensions (width and depth) of example channel-forms in the outcrop. These measurements are also reported in Table 1.

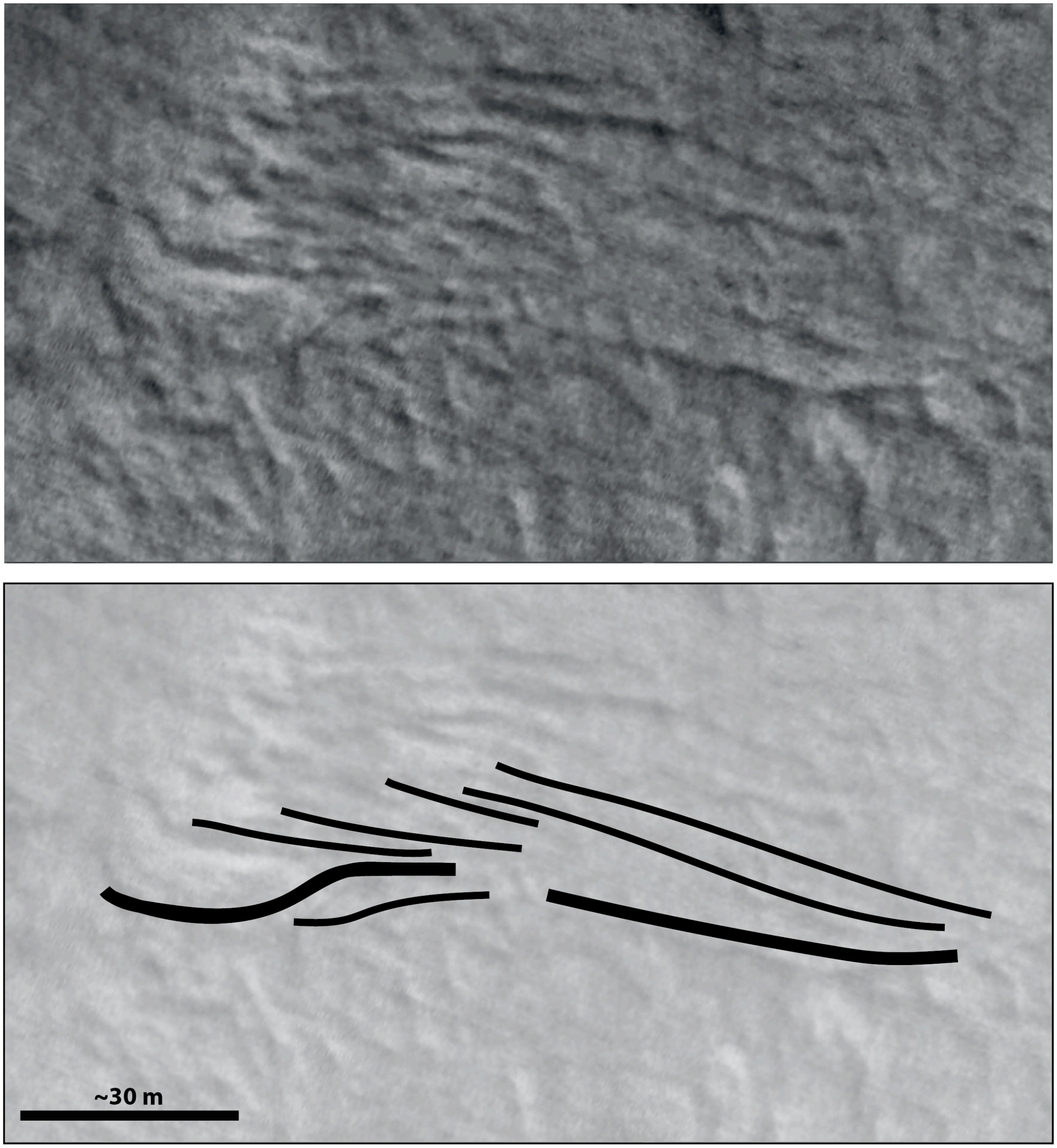

Supplementary Figure 3. Close up of a possible channel wing marked with an asterisk in Figure 2B.

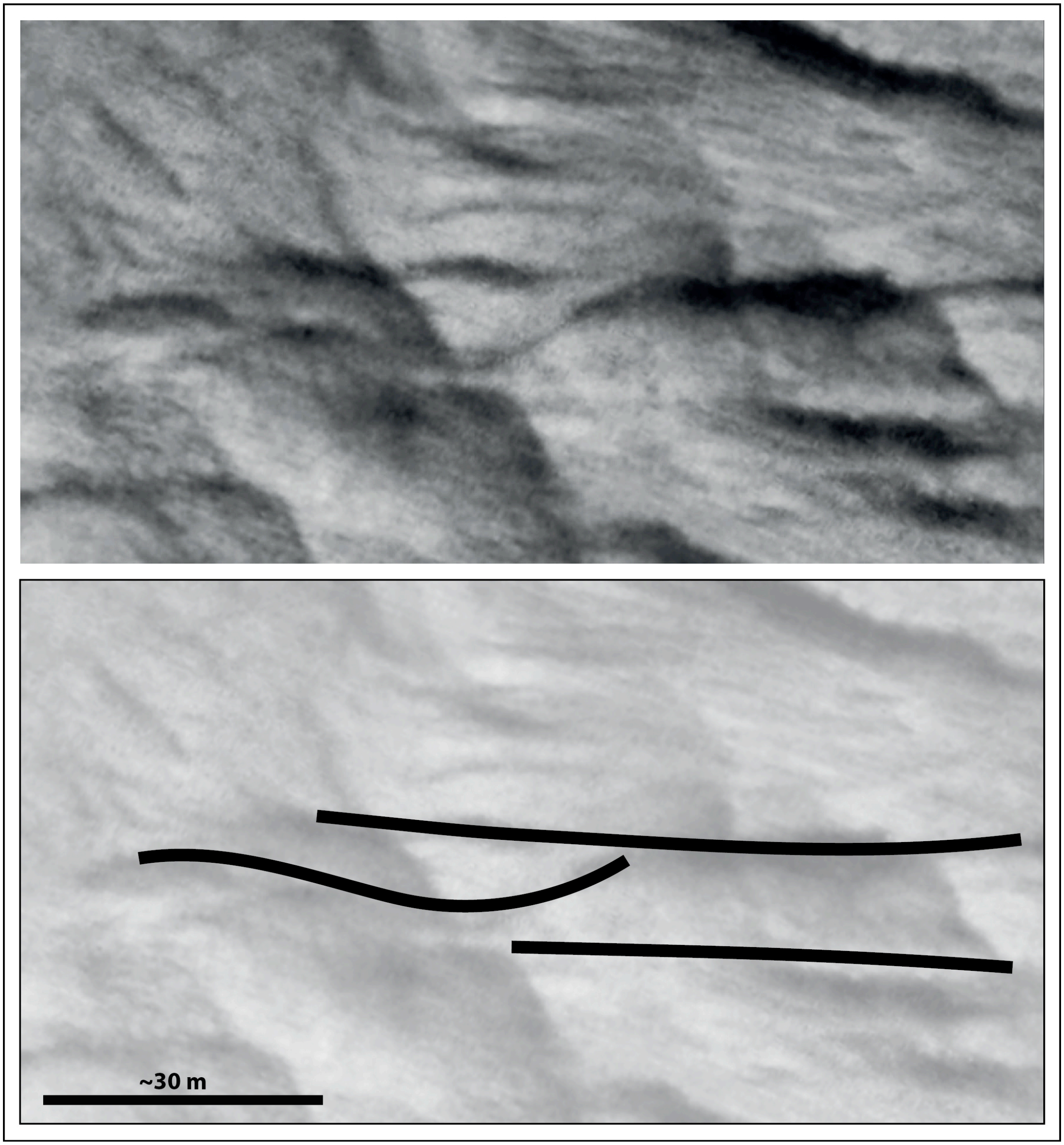

Supplementary Figure 4. Close up of a possible channel wing marked with a hash-tag in Figure 2B.

A

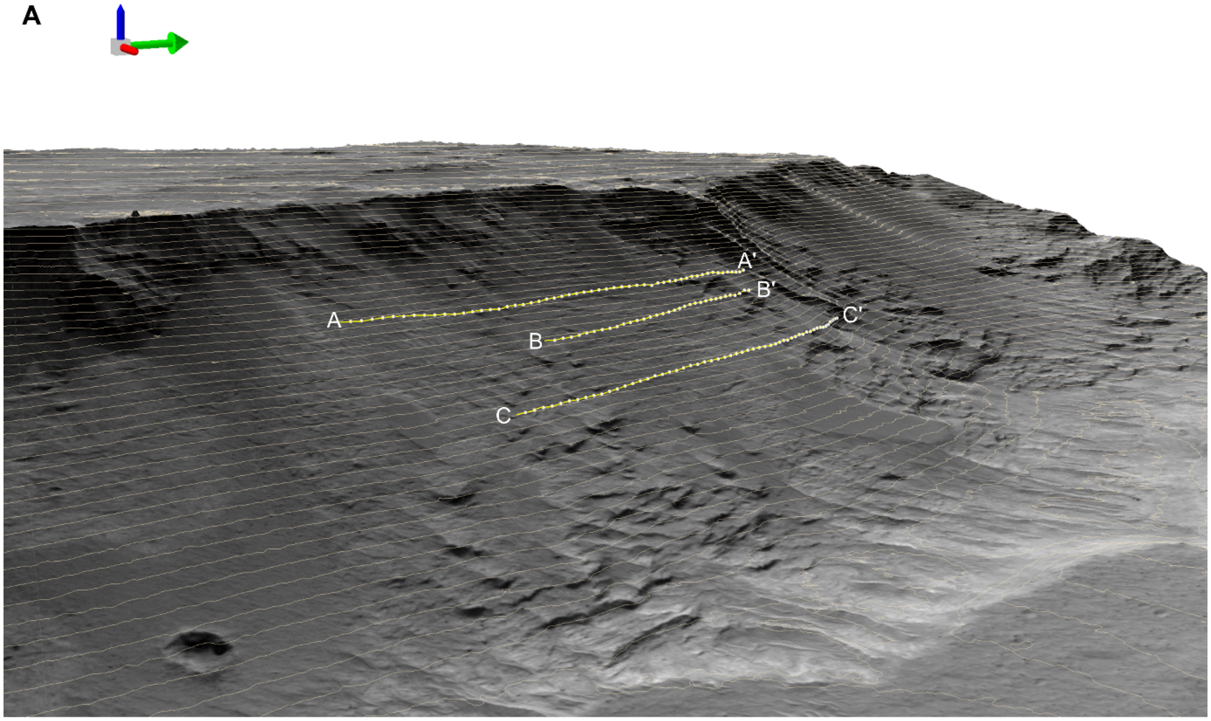

B

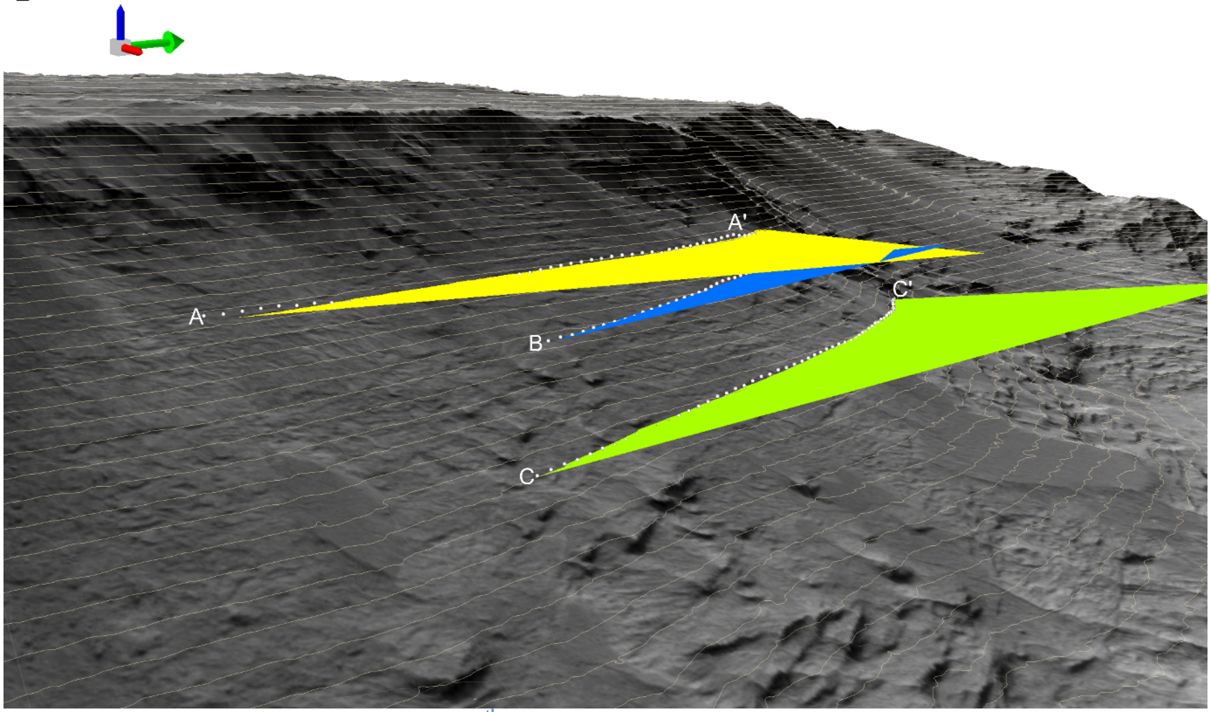

Supplementary Figure 5. Estimates of dip for 5th order surfaces. (A) 5 m spacing contour lines and digitization of the uppermost three 5th order surfaces. DEM sampling points are spaced every 10m. AA' is ~ 540 m long, Bb' is ~ 335 m long, and CC' is ~ 580 m.(B) The second image shows a linear trend fitted to the sampling points for each surface. For the topmost AA' surface, the dip is ~ 0.7° to the SSW. The BB' and CC' trends are similar: 10-11° to the SW. This low dip means that the measured thicknesses of strata will be within a few percent of those measured assuming a zero dip. The RMS (root mean square) vertical deviation of the input points from the calculated best-fit plane is < 1 m for BB' and CC' and < 1.5 m for AA', suggesting the planes are a good fit to the points. The similar dip direction and dip value of all three planes also provides confidence that the inferred dips are reliable. The green arrow point North.
